# Supplementary material for: Communication with Family and Friends across the Life Course
Source: PLoS One. 2016 Nov 28;11(11):e0165687. doi: 10.1371/journal.pone.0165687 (PMC5156499; doi:10.1371/journal.pone.0165687)
Supplement: S1 File — (DOCX) [file pone.0165687.s001.docx]

**Supporting Information for**

**Communication with family and friends across the life course**

Tamas David-Barrett, Janos Kertesz, Anna Rotkirch, Asim Ghosh, Kunal Bhattacharya, Daniel Monsivais, Kimmo Kaski

**Phone calls with three generations**


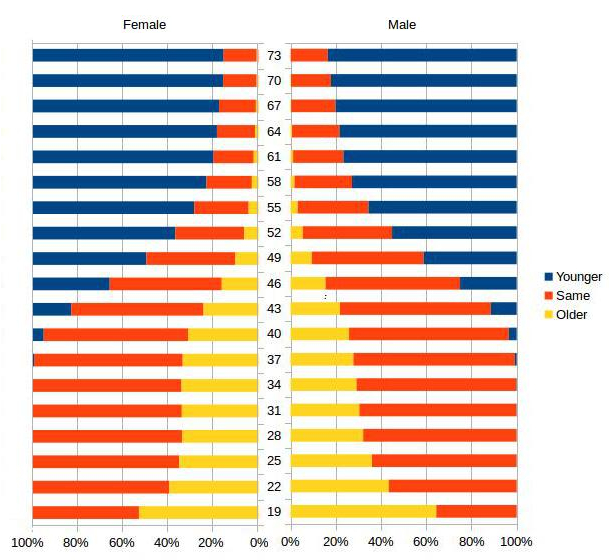


Figure A. Histogram of the fraction of the calls with the three generation by female egos (left panel), and by male egos (right panel), as a function of their age (middle panel). Blue, red, and yellow represent the generation of the alters, respectively: one generation younger alter, same generation alter, one generation older alter.

**Gender imbalance in the data**


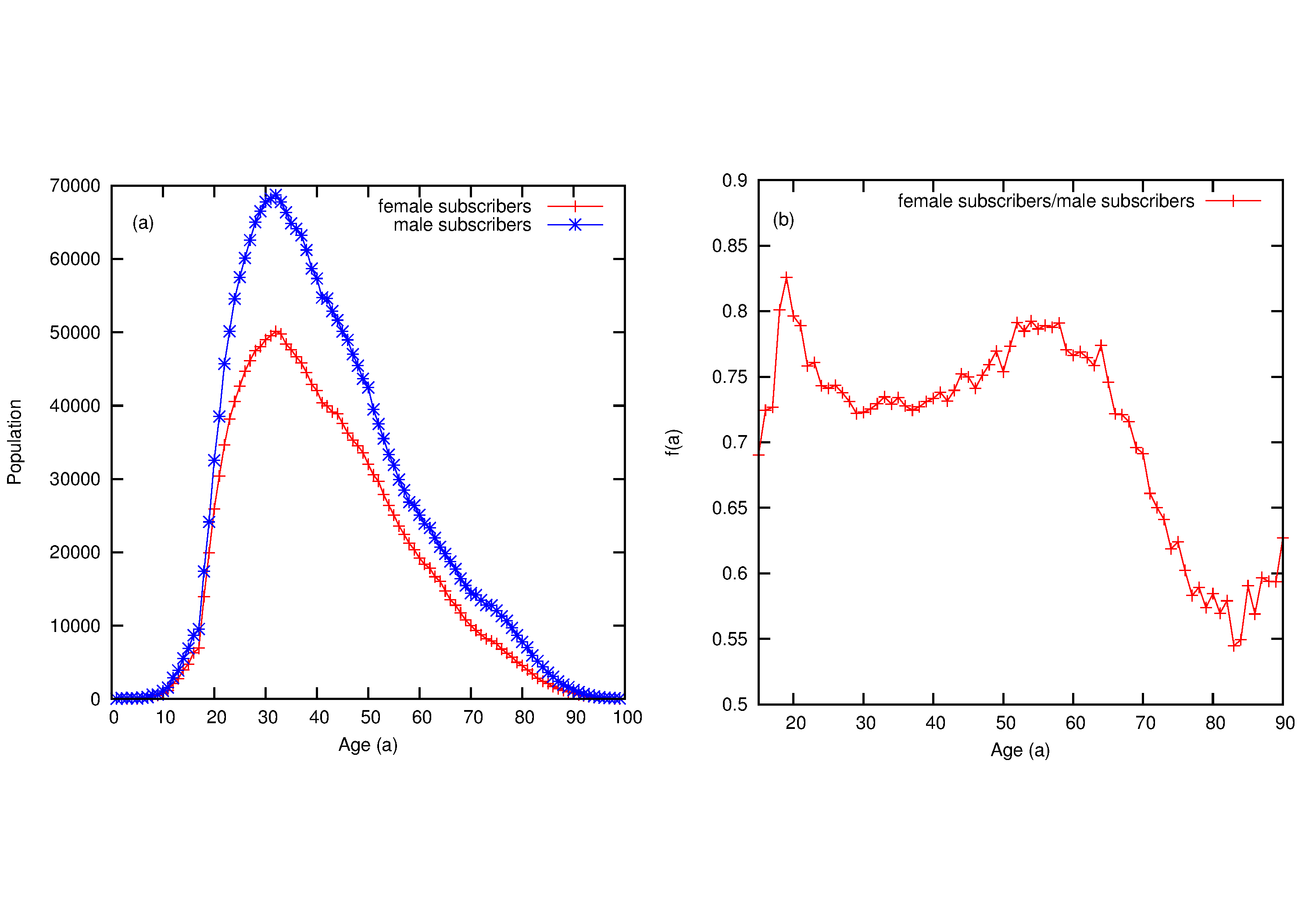


Figure B. Frequency difference between female and male callers in the database. Panel (a): histograms, panel (b): ratio between the two genders as a function of age.
